# The Significant Association between Polymicrobial Diabetic Foot Infection and Its Severity and Outcomes

**DOI:** 10.21315/mjms2019.26.1.10

**Published:** 2019-02-28

**Authors:** Sharifah Aisyah Syed Hitam, Siti Asma’ Hassan, Nurahan Maning

**Affiliations:** 1 Department of Medical Microbiology and Parasitology, School of Medical Sciences, Universiti Sains Malaysia, 16150 Kubang Kerian, Kelantan, Malaysia; 2 Hospital USM, Health Campus, Universiti Sains Malaysia, 16150 Kubang Kerian, Kelantan, Malaysia; 3 Pathology Department, Hospital Raja Perempuan Zainab 2, 15586 Kota Bharu, Kelantan, Malaysia

**Keywords:** diabetic foot infection, diabetes mellitus, microorganisms, polymicrobial infection, severity of infection

## Abstract

**Background:**

Foot infection is a major complication of diabetes mellitus (DM) and its agents are usually polymicrobial. This study aims to describe the agent and determine the association between polymicrobial infections and the severity of diabetic foot infections (DFI) and their outcomes.

**Methods:**

This retrospective cohort study was conducted during one year and it involved 104 patients. Their records were reviewed and assessed. The causative agents and its sensitivity pattern were noted. The results were presented as descriptive statistic and analysed.

**Results:**

A total of 133 microorganisms were isolated with 1.28 microorganisms per lesion. The microorganism isolated were 62% (*n* = 83) GN (Gram-negative) and 38% (*n* = 50) GP (Gram-positive). GN microorganisms include *Pseudomonas* spp (28%), *Proteus* spp (11%), *Klebsiella* spp (8%) and *E. coli* (4%). *Staphylococcus aureus* (54%) was predominant among GP, followed by Group B *Streptococci* (26%) and *Enterococcus* spp (6%). Thirty patients (28.8%) had polymicrobial infections. The association between the quantity of microorganisms and severity of DFI was significant. Among severe DFI cases, 77.8% with polymicrobial microorganisms underwent amputation compared to 33.3% with monomicrobial infection.

**Conclusion:**

GN microorganisms were predominantly isolated from DFIs and remained sensitive to widely used agents. Polymicrobial infections were associated with DFI severity.

## Introduction

Foot infections are among the most common lower extremity complications in the diabetic population (excluding neuropathy), second only to foot ulcers in frequency (1). As the incidence of diabetes mellitus (DM) is increasing globally, complications related to this endocrine disorder are also mounting and diabetic foot infections (DFIs) is an important cause of morbidity and mortality in patients with DM. DFI affect one in 10 patients with DM during their lifetime (2). They have increased risk of lower extremity amputations and the main cause is diabetic peripheral arterial disease accelerated by the direct damage to the nerves and blood vessels by high blood glucose levels. Smokers, older patients with a longer history of uncontrolled diabetes, and those with gangrenous infections and large ulcers have poorer outcomes with amputations (3).

It is well documented that diabetic foot infections are frequently polymicrobial in nature (4, 5, 6). Hyperglycemia, impaired immunologic responses, neuropathy, and peripheral arterial disease are the major predisposing factors leading to limb-threatening DFI (4).

Foot infections in persons with DM are often initially treated empirically. The empirical antibiotics used are usually meant for broad-spectrums organisms coverage or according to local antibiogram study. Hence, therapy directed at known causative organisms may improve the outcome. Many studies have reported on the bacteriology of DFIs over the past 25 years, but the results have varied, and they have often been contradictory (7). Therefore, study on the local causative organisms and antibiograms of DFI is an essential tool for better management of diabetic foot patients.

A number of studies have found that *Staphylococcus aureus* is the main causative pathogen (8, 9), but more recent investigations have reported a predominance of Gram-negative (GN) (10, 11). The role of anaerobes is especially unclear, because in many studies, specimens were not collected or cultured properly to recover these organisms. Among those that did use appropriate methods, some have reported that anaerobes play a minimal role and *Bacteroides fragilis* is the predominant anaerobe isolated (12, 13). These discrepancies of aetiological agents could be partly due to differences in the causative organisms occurring over time, geographical variations or the types and severity of infection included in the studies.

Optimal management requires aggressive surgical debridement and wound management, effective antibiotic therapy, and correction of metabolic abnormalities mainly hyperglycaemia and arterial insufficiency. In current practice, little attention is paid to the possible pathogen that causing the DFI although some pathogens have different types of virulence, as well as responding to different antibiotics. Polymicrobial infection of DFI also contributes to the severity of disease; therefore, it can be one of the prognostic factors and more vigilant management should be taken. This study describes the microorganisms involved in DFI, the association of polymicrobial infection with severity of infections and its outcome in term of amputation and discharge from hospital.

## Materials and Methods

This was a retrospective study that was carried out for a duration of one year from June 2014 to June 2015. All patients diagnosed as type 1 or type 2 DM in the ward and clinic of Hospital Universiti Sains Malaysia who were suspected of having DFI with or without bone infection based on clinical signs and symptoms using Infectious Disease Society of America (IDSA) (2) were included in this study. Patients who suffered from trauma or had incomplete details and more than 10% incomplete data records for the main variables of interest were excluded. Repeat patients with same diagnosis were also excluded. The data were collected from medical record obtained from the hospital record office. Relevant information on diabetic patients also has been explored. This information included patient demographic data like age and gender; comorbid illness like hypertension, ischaemic heart disease, bronchial asthma and chronic obstructive lung diseases (COAD); severity of the illness, diagnosis during admission; and laboratory result parameters (red blood cell count, haemoglobin level, total white cell counts and HbA1c). The sampling method applied in this study was a simple random sampling. During the data collection, the registration number of all patients that were admitted to the ward and clinic of Hospital Universiti Sains Malaysia were coded and kept separately with a confidential safe guard. Sample size for the association between polymicrobial infections and severity of DFI was calculated using a two proportion formula and the sample size was 104.

Regarding the selection of microorganisms, the data were extracted from the USM WHONET system. The quantity of microorganism was recorded. Monomicrobial infection was defined as a single infectious agent, whereas polymicrobial infections is a multiple infectious agents (14). All of the microorganism types including Gram-positive (GP), Gram-negative (GN) and anaerobes were recorded and analysed.

The data from patients’ medical records was extracted and recorded in Microsoft Office Excel 2013. After reviewing the data, all the relevant information was entered into IBM SPSS Statistics Version 22. The statistical analysis that was used in this study was descriptive and categorical data analysis (Fisher’s Exact test or Pearson Chi-square test). The descriptive analyses were performed for both numerical and categorical independent variables. The measurements used for the numerical independent variable were the mean and standard deviation (SD). The statistic could also be reported as the median and inter-quartile range (IQR) when the normality distribution was skewed. Meanwhile, the frequency and percentage were examined for the categorical independent variable. A *P*-value with less than 0.05 indicated statistically significant. The approval from Research Ethics Committee (Human) USM (JEPeM) were obtained (Ref. No.:15010011).

## Results

A total of 104 DFI patients who fulfilled the inclusion criteria were successfully recruited. The gender distribution among the patients with DFI was almost equal at 44% (*n* = 46) male and 56% (*n* = 58) female. Most of the DFI patients were aged 41–60 years old. There were two younger patients with DFI at the ages of 25 and 27 years old. Both of them had type 1 DM.

A total of 133 microorganisms were isolated. Among them, the commonest were *Pseudomonas* spp, followed by *Staphylococcus aureus* (Table 1). The antibiotic sensitivity patterns of the GP and GN organisms are shown in Table 2 and Table 3.

Thirty patients (28.8%) had polymicrobial infections and 74 (71.2%) patients had monomicrobial infections. There were statistically significant between two interested variables of interest with a *P*-value less than 0.05. Fisher’s Exact test (*P* = 0.003), as shown in Table 4. Patients with polymicrobial DFI had higher glucose levels and total white cells. In contrast, the haemoglobin was found to be lower (Table 5).

## Discussion

DFI is often inadequately managed due to a lack of understanding of the microbial prevalence, antibiotic sensitivity and therapeutic approaches. This will lead to increased infection-related morbidity, increased duration of hospital stay and the incidence of major limb amputation.

The etiological agents of DFI are mostly due to GN bacteria. In this study, we found that GN bacteria are the predominant aetiological agents representing a percentage of 62.4% of the total isolates. Similar results have been found in many other studies worldwide (9, 10, 15). In contrast, studies from developed countries like those in North American and Europe found that GP aerobes especially *Staphylococcus aureus*, are the predominant pathogens in DFI (7, 16). A large multicentre study from United States from 2008 revealed that 77% of DFI, were caused by GP aerobes, while 21.2% were caused by GN aerobes (17). Another study done in Turkey by Hatipoglu et al. (18) in 2014 found that both aerobic GP and aerobic GN organisms were isolated with almost equal frequency from DFIs, throughout period of 1989–2011 (48.4% versus 48.4%) (18).

Among GN bacteria isolated in this study, the most common were *Pseudomonas* spp (27.8%), followed by *Proteus* spp (10.5%) and *Klebsiella* spp (8.3%). Similar results were recorded by Ramakant et al. in 2011, Hatipoglu et al. in 2014 and Hobizal et al. in 2012 (15, 18, 19). On the other hand, another study showed *E. coli* (20.3%) and *K. pneumoniae* (17.4%) were the leading cause of DFI (20). Among GP bacteria, *S. aureus* is the commonest isolate. The result is similar to those recent studies either from eastern or western region. (11, 15, 18, 20, 21).

It is well documented in the literature that DFIs are polymicrobial in nature (5, 7, 22). Our study observed that moderate to severe DFI comprised infection by polymicrobial organisms whereas mild DFI are mostly monomicrobial. The findings are similar to studies done by many other researches (4, 15, 23, 24). We found about 28.8% (*n* = 30) of DFI patients had polymicrobial infections which is represented a lower level than those found in other studies. The reason for this maybe because, in this study, the isolate was more towards aerobic culture, with the fact that polymicrobial infections in DFI contain anaerobic organisms. This study gained only two anaerobic organisms, which were both *Bacteroides fragilis. Bacteroides fragilis* is the commonest species of anaerobic organisms found in DFI (4, 9, 10, 25).

The microorganisms isolated in this study were sensitive to a number of agents. The carbapenem group including ertapenem, meropenem and imipenem, showed good activity toward GN bacteria except for *Acinetobacter* spp, which are usually multidrug resistant and only sensitive to polymixin B and colistin. Oxacillin had very good coverage for sensitive strains of *Staphylococcus aureus* and vancomycin was found to be the most effective drug overall against GP organisms. These findings were in accordance with previous studies (9, 26).

As stated above, *Pseudomonas* was the most common organism found in this study. Although isolated in many patients, it is often considered a non-pathogenic coloniser when isolated from wounds. Of all *Pseudomonas* spp, *P. aeruginosa* was isolated in less than 10% of wounds (27, 28). The suggested empirical antibiotic regimens for treatment of DFI for patients with risk factors for polymicrobial infection include ampicillin/sulbactam (unasyn), ceftriaxone plus clindamycin or metronidazole, levofloxacin plus clindamycin, moxifloxacin and ertapenem (29).

Clinicians must also consider covering other resistant organisms, including extended spectrum beta lactamase producing Gram negative isolates and methicillin resistant *Staphylococcus aureus* especially in countries in which they are relatively common (30). Other laboratory parameters are also important in diagnosing and managing DFI. Another study found that, severe DFI patients with polymicrobial infections had higher glucose levels and total white cell counts. In contrast, the haemoglobin was found to be lower. In physiological terms, a higher blood hemoglobin level indicated that more oxygen molecules were carried to local tissue, and consequently, anabolism and catabolism occurred more effectively. Blood haemoglobin is also a good indicator of nutritional status. The above two factors were probably the reason why low blood haemoglobin levels are found in DFI (31). An elevated white blood cell (WBC) count might reflect responses to both inflammation and infection, and it could be an important risk factor for amputation in DFI (32).

In general, there were many outcomes of polymicrobial infection. However, this study only focused on amputation and discharge in individual patients. Our study showed that the outcomes exhibited no statistically significant different between amputation and discharge of patients with severe polymicrobial DFI. In a few studies, the authors described individual pathogens to assess predictive factors for limb loss rather than comparing polymicrobial and monomicrobial infections; for example, they found that DFI infected with *Staphylococcus aureus* (*P* = 0.042) was predictive factor for limb loss (33). Severity of DFI can be confounded by other factors such as age of patients, duration of DM, control of DM and so on. Interactions with all these factors may be present. Therefore, all these factors must also be considered apart from polymicrobial infections for the determination of severe DFI.

In conclusion, GN bacteria are dominant in DFI patients. *Pseudomonas* ssp. and *Staphylococcus aureus* were the most commonly identified GN and GP microorganism. The sensitivity patterns of common organisms suggested that they are susceptible to commonly use drugs. Polymicrobial infections were found in 28.8% of subjects and this was associated with severity of DFI. Conversely no such association was seen in limb amputation and discharge of patients with severe polymicrobial infection.

## Figures and Tables

**Table 1:** Distribution of aetiologic agent in DFI participants (*n* = 133)

Pathogen	*n* (%)
Gram positive	
• *Staphylococcus aureus*	27 (20.3)
• *Streptococcus agalactiae*	13 (9.8)
• *Enterococcus* spp	4 (3.0)
Other Gram positive organisms	6 (4.5)
Gram negative	
• *Klebsiella* spp	11 (8.3)
• *Escherichia. coli*	5 (3.8)
• *Proteus* spp	14 (10.5)
• *Enterobacter* spp	8 (6.0)
• *Pseudomonas* spp	37 (27.8)
• *Acinetobacter* spp	6 (4.5)
Anaerobes	2 (1.5)

**Table 2:** Antibiotic sensitivity pattern of Gram-positive pathogen (*n* = 50)

Antibiotic	*Staphylococcus aureus* *n* (%)	*Streptococcus agalactiae* *n* (%)	*Enterococcus* spp *n* (%)	*Other organism *n* (%)
Oxacillin	26 (96.3)	-	-	-
Penicillin	5 (18.5)	13 (100.0)	4 (100.0)	6 (100.0)
Gentamicin	27 (100.0)	13 (100.0)	4 (100.0)	6 (100.0)
Rifampicin	27 (100.0)	-	-	-
Vancomycin	27 (100.0)	-	4 (100.0)	-
Trimethoprim sulfamethoxazole	27 (100.0)	-	-	6 (100.0)
Clindamycin	27 (100.0)	-	-	-
Fusidic acid	24 (88.9)	-	-	-
Erythromycin	27 (100.0)	12 (92.3)	4 (100.0)	6 (100.0)

* *Streptococcus pyogenes, Streptococcus* group C*, Streptococcus* group G*, Streptococcus mitis* and coagulase-negative *Staphylococcus aureus*

**Table 3:** Antibiotic sensitivity pattern of Gram-negative microorganisms (*n* = 83)

Antibiotic	*Klebsiella* spp *n* (%)	*E. coli* *n* (%)	*Proteus* spp *n* (%)	*Enterobacter* spp *n* (%)	*Pseudomonas* spp *n* (%)	*Acinetobacter* spp *n* (%)	Anaerobes* n (%)
Ampicillin	0 (0)	2 (40)	8 (57)	1 (17)	-	-	-
Gentamicin	11 (100)	5 (100)	12 (86)	8 (100)	-	0 (0)	-
Amikacin	11 (100)	5 (100)	14 (100)	8 (100)	-	0 (0)	-
Cefuroxime	8 (73)	3 (60)	12 (86)	8 (100)	-	-	-
Cefotaxime	8 (73)	3 (60)	14 (100)	8 (100)	-	0 (0)	-
Ceftazidime	8 (73)	3 (60)	14 (100)	8 (100)	36 (97)	0 (0)	-
Cefepime	8 (73)	3 (60)	14 (100)	8 (100)	35 (95)	0 (0)	-
Amoxycillin-clavulanate	8 (73)	5 (100)	7 (50)	2 (33)	-	-	-
Trimethoprim sulfamethoxazole	8 (73)	5 (100)	12 (86)	4 (67)	-	0 (0)	-
Ciprofloxacin	10 (91)	5 (100)	13 (93)	8 (100)	34 (92)	0 (0)	-
Piperacillin-tazobactam	11 (100)	5 (100)	14 (100)	8 (100)	34 (92)	0 (0)	-
Ertapenem	11 (100)	5 (100)	14 (100)	8 (100)	-	-	-
Meropenem	11 (100)	5 (100)	14 (100)	8 (100)	36 (97)	0 (0)	-
Imipenem	11 (100)	5 (100)	13 (93)	8 (100)	37 (100)	0 (0)	-
Metronidazole	-	-	-	-	-	-	2 (100)
Polymyxin b	-	-	-	-	-	6 (100)	-
Colistin	-	-	-	-	-	6 (100)	-

* Bacteroides fragilis

**Table 4:** Associated factors between quantity of microorganisms among patients and severity of DFI (*n* = 104)

Quantity of Organisms	Severity of diabetic foot infection			*P*-value

	Mild *n* (%)	Moderate *n* (%)	Severe *n* (%)	
Monomicrobial	14 (17.7)	59 (74.7)	6 (7.6)	0.003^a^
Polymicrobial	2 (8.0)	14 (56.0)	9 (36.0)	

a Fisher’s Exact test was applied

**Table 5:** Other parameters for polymicrobial DFI (*n* = 30)

Variable	Polymicrobial DFI

	Mean (SD)
HbA1c	11.8 (2.1)
RBS	16.3 (5.2)
TW	16.6 (2.4)
Haemoglobin	9.0 (1.3)
